# Pearls in running drops on an inclined glass substrate excited by Lamb waves

**DOI:** 10.1038/s41598-017-14662-9

**Published:** 2017-10-26

**Authors:** Wei Liang, Sabrina Tietze

**Affiliations:** 1 Automotive Engineering College, Shanghai University of Engineering Science, Shanghai, 201620 China; 2 Institute of Sensor and Actuator Technology, Coburg University of Applied Sciences and Arts, Coburg, 96450 Germany

## Abstract

We demonstrate that pearling droplets will be released from droplets as they sliding down a partially wetting glass plate excited by Lamb waves. During the movement, we find that the transitions at generating pearling are independent of the drop size and depend only on a critical capillary number *Ca*. Further up, the position of the pearls must be at or around the droplet’s advancing or receding end of the initial state.

## Introduction

**Figure 1:**
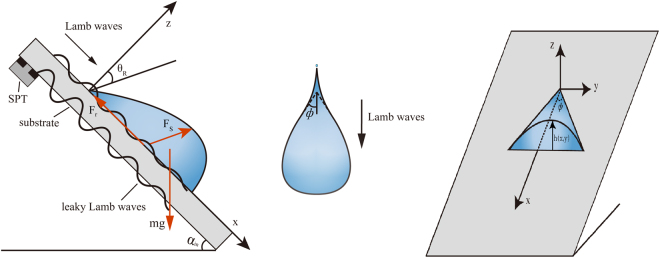
Schematic diagram of mode conversion of Lamb waves into compressional sound waves at the liquid-solid boundary of the droplet resulting in leaky Lamb waves on an inclined substrate at an inclination angle of α_in_. (**a**) Side view of a drop running down a plate, (**b**) notation *Φ* is the half angle of the receding contact line in macroscopic region, (**c**) the structure at the rear of sliding drop.

A liquid drop on an inclined non-piezoelectric substrate, on which Lamb waves are propagating, will slide downward very fast due to acoustic streaming force and gravity. A study of this work is not only interesting but has applications in many practical areas such as removal of water droplets from the windscreen of a car or from optical devices^1^. But the removal of droplet excited by Lamb waves, always causes the smaller drops (pearls) sticking on the surface. Perhaps unexpectedly, this problem has received rather little attention. Available studies mostly focused on motion of drop propelled on a horizontal substrate excited by Lamb waves (solely driven by acoustic streaming force) without concerning the pearls deposition^2^, or static/quasi-static drops^3–5^, or electrically assisted drop sliding on inclined planes without concerning the pearls deposition^6^, or motion and shape of drops sliding on a sloping substrate (sole driven by gravity)^7–10^. Podgorski *et al*.^7^ and Grande *et al*.^8^ demonstrated that above critical capillary number, drops exhibit a cusped tail which emits pealing drops. Those investigations were performed with gravity as the driving force. In the present work an additional force – acoustic streaming force resulting from Lamb waves on the substrate is applied. And both deformation of the droplet as well as pearlings are observed as well. We demonstrate the pearls emitted by the drop, which is propelled on a glass plate inclined at a variable angle α_in_ by applying Lamb waves on the substrate (see Fig. 1).

## Results

**Figure 2:**
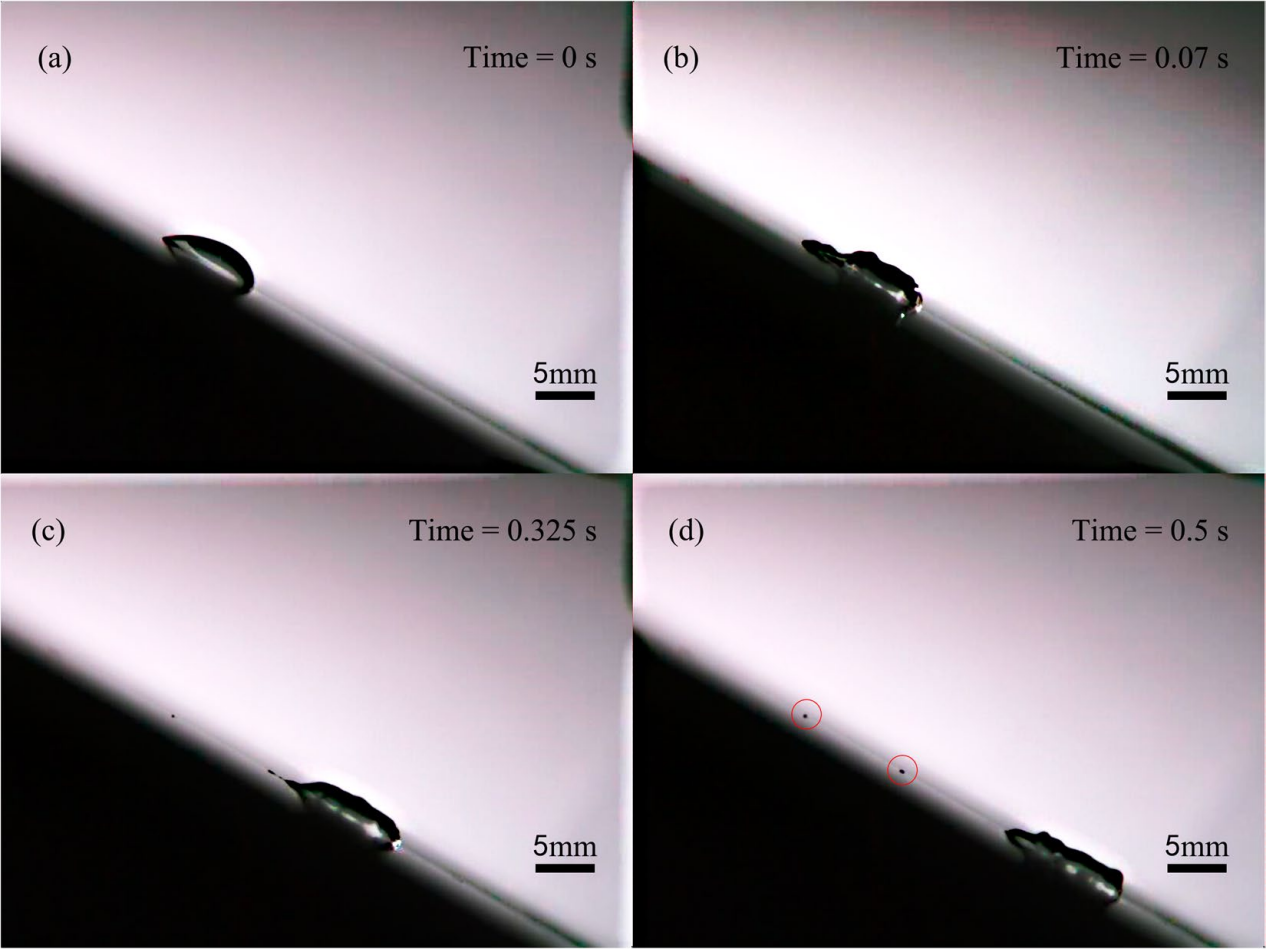
Experimental observation for a 30 μl droplet placed on a 1 mm thick inclined glass plate at an inclination angle of 30° at a wave amplitude of 17.95 nm (measured by laser-Doppler vibrometry) produced by a 1 MHz single phase transducer (SPT) at an input AC voltage 95 V_pp_. The observations are snapshots from video sequences recorded with a high-speed camera. The waves are propagating from left to right. (**a**) The initial state was at 0 s, (**b**) the droplet first becomes in a longitudinal expansion at the bottom, (**c**) above a critical speed, the trailing end breaks up to a pearl, (**d**) two pearl drops are pinned on the glass plate.

We placed droplet on the inclined substrate and used Lamb waves in our experiment, the droplet obtained acceleration along the x-axis and would slide down the plate eventually. But during the movement, the drop appears as pearl drops running along a line of quasi-static smaller droplets left by the previous drop. These smaller droplets (pearls) are pinned on the glass plate (Fig. 2(d). If we observe the droplet movement slides along the inclined solid surface in detail, as shown in Fig. 2, we will find that the droplet first becomes in a longitudinal expansion at the bottom because of the acoustic streaming force, the resistance force and also the gravity (Fig. 2(b). Above a critical speed the trailing end breaks up to a pearl (Fig. 2(c). That is because the singularity emerges on the sharp end of droplet, the singular point can become unstable and emit pearls entrained by the tape^7^. Finally, the pearls are pinned on the glass plate and vibrate (Fig. 2(d).

We record the pearl drops for different droplet volumes (10 μl, 20 μl, 30 μl) at different inclinations of the plate α(30°, 45°, 60°). The maximum length L of the droplets before breakup were measured, as shown in Table 1, which could drastically increases in the pearl regime^8^.

**Table 1:** The maximum length L of the droplets before breakup for droplets with volume of 10 μl, 20 μl, and 30 μl at inclinations of the plate of 30°, 45°, and 60°. The symbol ‘*’ denotes the exceptional case that there are three pearl drops left from the previous droplet which are dropped simultaneously at or around the position of droplet’s advancing end of initial state.

Volume(μl)	α_in_(°)	Initial length of the droplet (mm)	Length of the droplet (before the first pearl dropped down) (mm)	Length of the droplet (before the second pearl dropped down) (mm)
10	30	8.63	9.11 ± 0.5	/
20	30	9.62	13.32 ± 0.5	/
30	30	8.73	12.375 ± 0.5	12.28 ± 0.5
10	45	7.13	11.352 ± 0.5	9.44 ± 0.5
20	45	8.27	15.90 ± 0.5	12.59 ± 0.5
30	45	9.67	15.90 ± 0.5	/
10	60	8.31	16.51 ± 0.5	/
20	60	9.89	14.62 ± 0.5	/
30	60	9.07	23.48* ± 0.5	/

**Figure 3:**
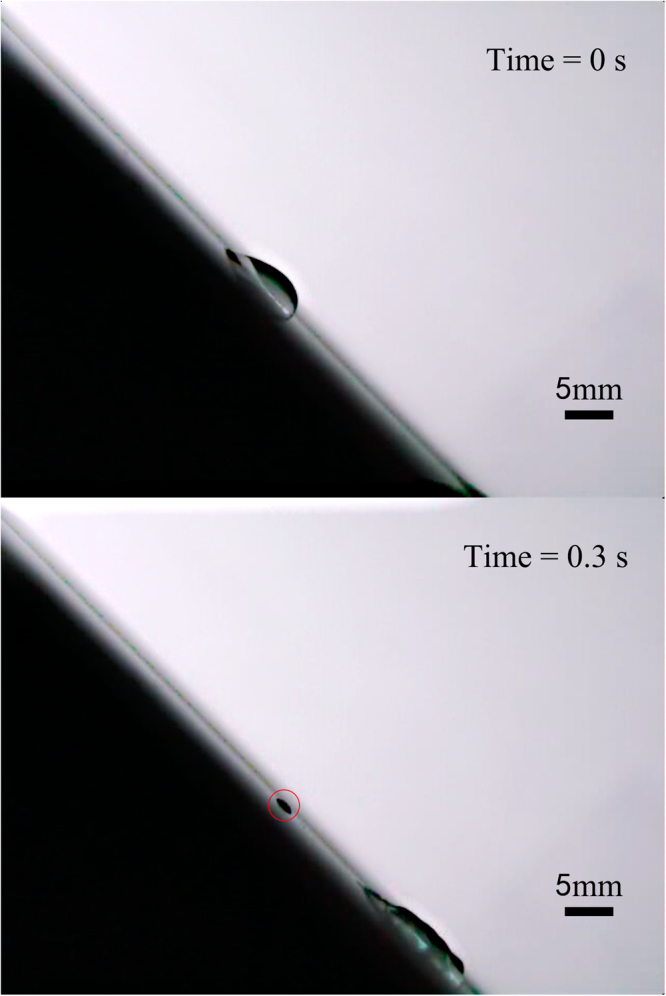
30 μl droplet movement induced by 1 MHz Lamb waves at inclination of the plate 45°. The place of the dropped pearls is at the position of droplet’s advancing end of initial state.

**Figure 4:**
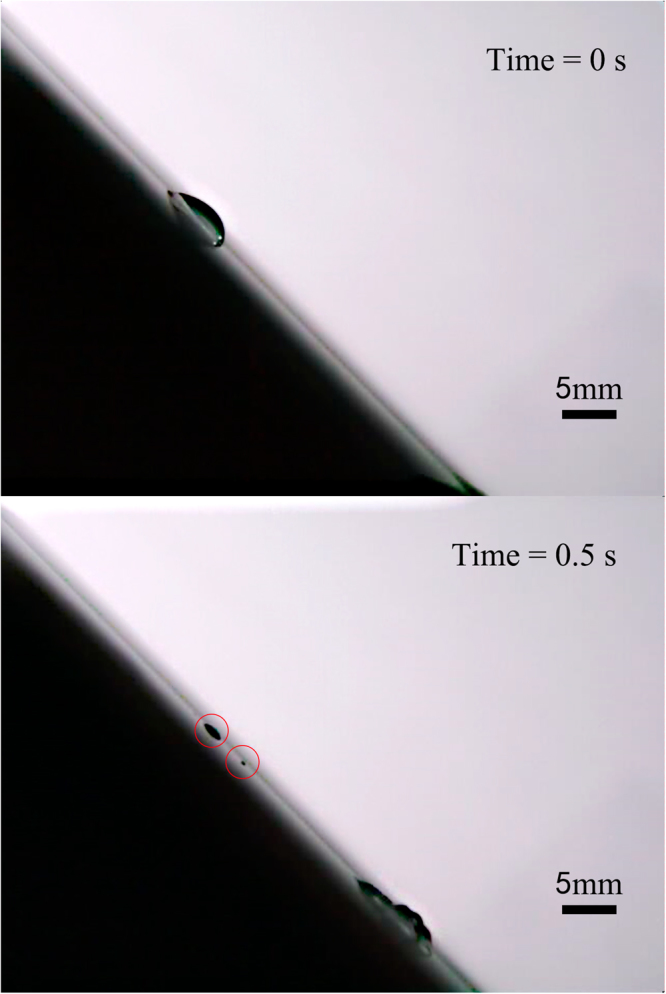
10 μl droplet movement induced by the 1 MHz Lamb waves at an inclination angle of 45°. The first pearl was dropped at the position of droplet’s advancing end of initial state, the second pearl followed immediately the first one within 0.02 s.

**Figure 5:**
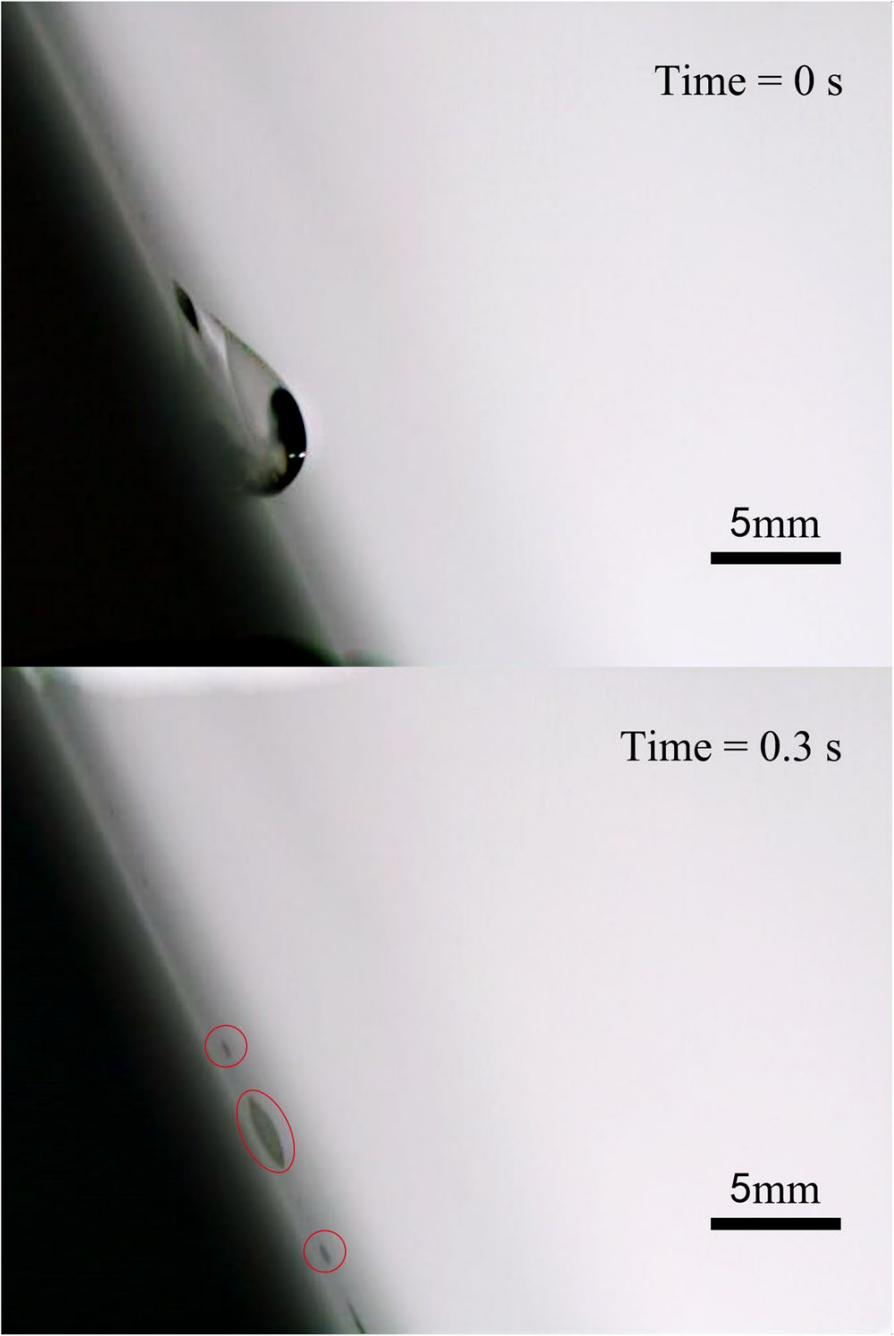
30 μl droplet movement induced by the 1 MHz Lamb waves at an inclination angle of 60°. The first and third pearls were dropped near the position of droplet’s advancing end of initial state; the second pearl was dropped just at the position of droplet’s advancing end of initial state.

In the experiment on an area of 20 cm × 20 cm glass plate, we found that there were maximum two pearls could be separated from the droplet, except the measurement for droplet volume of 30 μl at an inclination angle of 60°. The most important observation is that for all measurements the pearls were always generated at the position of droplet’s advancing end of initial state (Fig. 3). Especially, for droplet with volume of 30 μl at an inclination angle of 30°, when the droplet slided down the plate induced by Lamb waves and gravity, the first dropped pearl was around the position of droplet’s receding end of initial state, the second pearl was around the position of droplet’s advancing end of initial state, as shown in Fig. 2. For the droplet volume of 10 μl at an inclination angle of 45°, the first pearl was dropped at the position of droplet’s advancing end of initial state, the second pearl followed immediately the first one within 0.02 s, as shown in Fig. 4. The most interesting phenomenon was for the droplet volume of 30 μl at an inclination angle of 60°, there were three pearls separated from the droplet simultaneously at or around the position of droplet’s advancing end of initial state, as shown in Fig. 5. Meanwhile, the length of the droplet before the second pearl dropped down could be shorter than the first one, which can also imagine in the reality that the volume of the remainder droplet is smaller; therefore the length of the droplet may get a little bit shorter.

## Discussion

**Figure 6:**
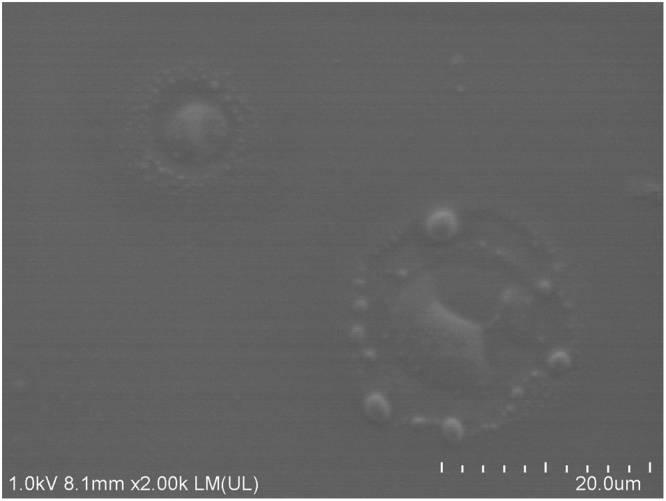
The SEM (Scanning Electron Microscope) image of the surface.

When the input power on the SPTs is immediately on, at this moment the velocities at liquid-solid interface are changing gradually except the positions around droplet’s advancing and receding ends^15^. Because of the inertia, pearls could be most probably left in the grooves (owing to surface roughness shown in Fig. 6) around the positions of droplet’s advancing and receding ends of initial state.

Meanwhile, it is generally observed that at the same inclination of substrate the receding angles decrease with increasing volumes of droplet; at the same volume of droplet the receding angles decrease with increasing inclinations of plate (Table 2). That is because of the gravity, under a certain inclination angle, the larger the droplet volume, the larger the advancing angle and the smaller the receding angle. Therefore under a certain inclination angle, a large contact angle hysteresis for large droplets are showed in Table 2.

**Table 2:** Critical capillary number for pearl generation and number of pearls in the experiments.

Volume(μl)	${{\boldsymbol{\alpha }}}_{{\bf{in}}}$(°)	θa (°) initial state	θr (°) initial state	Number of pearls	U (m/s) (before breakup)	Ca-critical
**10**	**30**	70 ± 2	38 ± 2	1	0.0605	0.000833
**20**	**30**	64 ± 2	37 ± 2	1	0.055966	0.00077
**30**	**30**	74 ± 2	36 ± 2	2	0.05799	0.000798
**10**	**45**	72 ± 2	37 ± 2	2	0.059417	0.000818
**20**	**45**	74 ± 2	34 ± 2	2	0.059496	0.000819
**30**	**45**	78 ± 2	33 ± 2	1	0.05773	0.000795
**10**	**60**	71 ± 2	28 ± 2	1	0.055106	0.000759
**20**	**60**	77 ± 2	26 ± 2	1	0.06	0.000826
**30**	**60**	86 ± 2	20 ± 2	3	0.056925	0.000784

**Figure 7:**
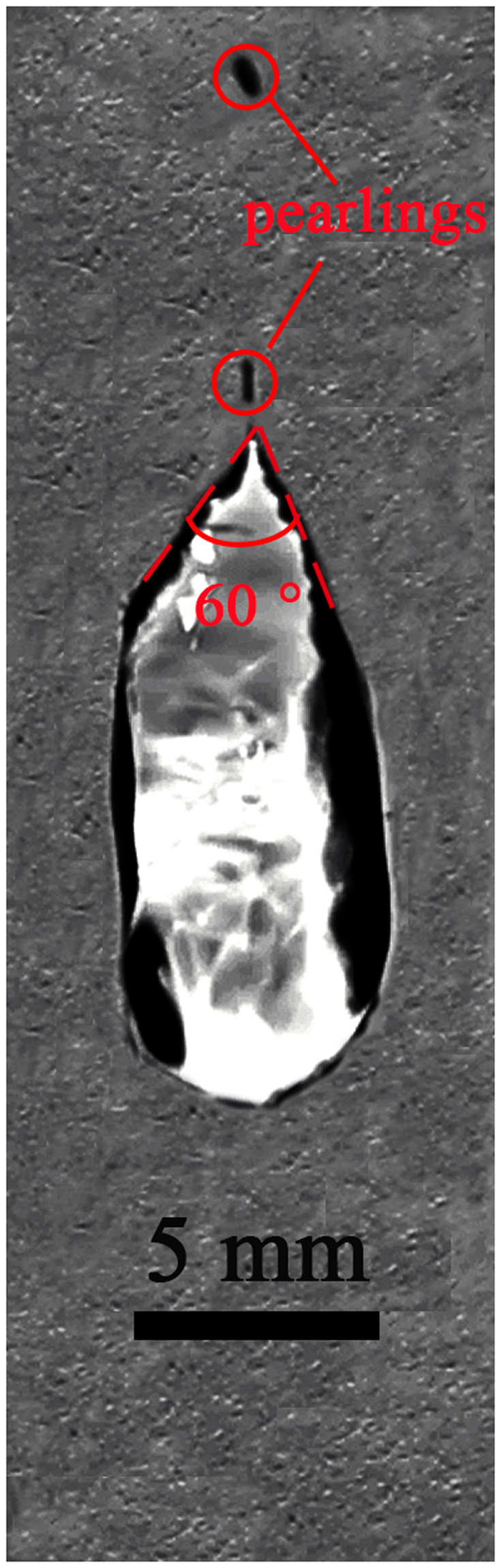
Pearling drop releasing from droplets at the angle Φ = 30°, which is the half angle of the indicating angle.

It is observed in Fig. 7 that because of the vibrations at the droplet surface the receding contact lines are asymmetric compared with the experiments of ref.^7^. Those vibrations are the capillary waves which don’t substantially contribute to the movements. Furthermore, the pearling generation is independent of the drop size and depend only on a critical capillary number *Ca*, where Ca = ηU/γ, U is the velocity of the fluid at the receding contact line,ηis the viscosity (the water viscosity at 20 °C is 1.005 mPa · s), γis the liquid–vapor interfacial tension(the water-vapor interfacial tension is 0.073 N/m). At low *Ca* (low speed) the tip curvature 1/R (R is the tip radius) stays nearly constant, while at the pealing generation (at high *Ca*/high speed) the curvature increases nearly 2 orders of magnitude, at which the singular point at the tip can become unstable and emit pearls entrained by the receding end^11^. For water drop motion by using Lamb waves in our case, the critical capillary number is about 0.0008 when the pearling generation occurs (Table 2). The critical capillary number for pearling without using Lamb waves is around 0.007^11^. That is because the velocity of the droplet at the receding contact line induced by Lamb waves sometimes is much more smaller than the other part of the droplet, or even the receding end could move in an opposite direction^12^. At these moments pearls are easily generated, therefore the critical capillary number at pearling generation using acoustic waves is much smaller than it without acoustic waves.

In ref.^7^, Podgorski *et al*. demonstrated that the pearls were released into periodic series. However, although in this work we trace the vibrating droplet in 40 mm, which is 4 or 5 times longer than the initial length of the droplet, obviously within this range we observe irregular series of pearls, and the number of generated pearls are shown in Table 2. We have shown that the position of the pearling drops induced by Lamb waves on an inclined glass substrate must be at or around the position of droplet’s advancing or receding end of initial state. This will allow the optimization of devices for droplet removal from windscreens or from optical mirrors, which may have the shortcomings that pearling drops releasing from the previous droplet could be stay on the substrate. Therefore, the detection of place of droplet will be the task in refined version of the droplet removal from windscreens or from optical mirrors. The surface acoustic waves could also be one of the choices on the droplet’s position detection.

## Methods

### Characterizations

The setup consists of a 20 cm × 20 cm × 1 mm glass plate with an inclination angle of α_in_ and a 1mm thick single phase transducer (SPT) attached to the opposite side of substrate (Fig. 1). This transducer consists of poled lead zirconate titanate (PZT) ceramics equipped with 2 metallic finger electrodes on one side and a metallic counter electrode on the other side, so called single phase transducer^13^. The wavelength of the Lamb waves are determined by the distance of the two electrode fingers of the SPT. With the 1 mm thick glass plate used in our investigations and a wavelength of 2.4 mm resulting from the two electrodes of the SPT Lamb waves were excited at a frequency of 1 MHz which results from the wavelength and the phase velocity of the Lamb waves on the substrate. Water droplets were loaded on the central axis of the propagating Lamb waves at an AC excitation voltage of 95 V_pp_, which will make the droplet to propel on the plate. The surface of the glass plate was covered by a hydrophobic layer (Rain-X). The hydrophobic layer was coated and allowed to dry. This process was repeated until to obtain a droplet contact angle of 90° when the substrate is horizontal. In the experimental investigations, because drops are transparent, it is hard to observe without appropriate lighting. With a piece of white paper behind the droplet, we fix a strong illumination gradient perpendicular to the direction of flow. Droplets act as focusing lenses and appear on the transparent glass plate. The motion was recorded via a high speed camera (Keyence VW900) and the frame rate is 4000 fps, which allows a time-resolved analysis of the droplet motion during propulsion.

### Theoretical analysis

There are three main forces when droplets move on an inclined non-piezoelectric substrate, namely, the acoustic streaming force *F*_*S*_, the gravity of droplet *mg*, and the hysteresis resistance force *F*_*r*_, as shown in Fig. 1. Acoustic streaming force is the leading force in the droplet propulsion induced by Lamb waves. Acoustic streaming force within a droplet results from the mode conversion of the Lamb waves into a compressional sound wave at the solid-liquid boundary, which is refracted at an angle θ_R_ termed Rayleigh angle or Lamb angle, are given in two dimension by^14^

1 $${F}_{Sx}=-\rho (1+{\alpha }_{1}^{2}){A}^{2}{\omega }^{2}{k}_{imag}\exp 2({k}_{imag}x+{\alpha }_{1}{k}_{imag}z)$$

2 $${F}_{Sz}=-\rho (1+{\alpha }_{1}^{2}){A}^{2}{\omega }^{2}{k}_{imag}{\alpha }_{1}\exp 2({k}_{imag}x+{\alpha }_{1}{k}_{imag}z)$$

where ρ is the density of the droplet, *ω* is the angular frequency, *A* is the amplitude of the displacement of Lamb waves from the inlet to the droplet, and *k*_imag_ is the energy dissipation of Lamb waves into the droplet. Here ${{\rm{\alpha }}}_{1}=-{\rm{j}}{\rm{\alpha }}$, $\alpha$ represents the attenuation constant, ${{\rm{\alpha }}}^{2}=1-{({{\rm{c}}}_{{\rm{s}}}{/c}_{{\rm{f}}})}^{2}$, where *c*_f_ is the sound velocity in liquid, *c*_s_ is the wave’s phase velocity. Since ${{\rm{c}}}_{{\rm{f}}\_{\rm{Water}}}$ = 1480 m/s and c_s_ = 2400 m/s are the sound velocity in water^15^ and the anti-symmetric zero mode of Lamb waves^12^ respectively, we could obtain the correlative attenuation constants in water, ${{\rm{\alpha }}}_{1\_{\rm{Water}}}=-{\rm{j}}\sqrt{1-({{\rm{c}}}_{{\rm{s}}}{/c}_{{\rm{f}}\_{\rm{Water}}})}=1.28$. As acoustic streaming force F_S_ is given by ${{\rm{F}}}_{{\rm{S}}}=\sqrt{{{\rm{F}}}_{{\rm{Sx}}}^{2}+{{\rm{F}}}_{{\rm{Sz}}}^{2}}$, we can derive the following form^14^

3 $${{\rm{F}}}_{{\rm{S}}}=-{\rm{\rho }}(1+{{\rm{\alpha }}}_{1}^{2}{)}^{\frac{3}{2}}{{\rm{A}}}^{2}{{\rm{\omega }}}^{2}{{\rm{k}}}_{{\rm{imag}}}\exp 2({{\rm{k}}}_{{\rm{imag}}}{\rm{x}}+{{\rm{\alpha }}}_{1}{{\rm{k}}}_{{\rm{imag}}}{\rm{z}}).$$

The direction of *F*_S_ is the same angle as the radiation of the leaky Lamb wave. Also, the propagation of acoustic radiation from a Lamb wave device into a droplet above it occurs through diffraction at the angle ${{\rm{\theta }}}_{{\rm{R}}}$. Thereby we can obtain the Rayleigh angle in water drop ${{\rm{\theta }}}_{\mathrm{R\_Water}}={\sin }^{-1}({{\rm{c}}}_{\mathrm{f\_Water}}/{{\rm{c}}}_{{\rm{s}}})\approx 38^\circ$.

Assume Lamb wave amplitudes are identical at the entrance points to drops for each measurement, with the same angular frequency *ω*, and using literature values of density ${{\rm{\rho }}}_{{\rm{Water}}}=1000\frac{{\rm{kg}}}{{{\rm{m}}}^{3}}$ ^15^, the leaky wave number in glass/water which represents attenuation is 95 m^−1^ calculated by the method of Rose^16^ and Lowe^17,18^, thereby the acoustic streaming forces in water and oil drops can be derived as

4 $${{\rm{F}}}_{{\rm{S}}\_{\rm{Water}}}=-4.1\times {10}^{5}\exp 2(95{\rm{x}}+121.6{\rm{z}}){{\rm{A}}}^{2}{{\rm{\omega }}}^{2},$$

The hysteresis resistance force, Fr, can be related to the contact angle hysteresis approximately through the equation^19^

5 $${{\rm{F}}}_{{\rm{r}}}\approx {\rm{\gamma }}w(\cos \,{{\rm{\theta }}}_{{\rm{r}}}-{\cos {\rm{\theta }}}_{{\rm{a}}})$$

here γ is the liquid–vapor interfacial tension, w is the width of the liquid-solid contact area, θ_a_ and θ_r_ are the advancing and receding angles respectively.

In the absence of Lamb waves, when the glass plate is inclined in the inclination angle of ${{\rm{\alpha }}}_{\mathrm{in}-{\rm{c}}}$, droplet remains at equilibrium on the inclined glass plate owing to the gravitational force attempting to deform the droplet and the force formed by contact angle hysteresis (CAH) is intended to prevent such drop motion. It is found that a drop in its critical configuration satisfies the relation^19^

6 $${\rm{mg}}\,{\sin {\rm{\alpha }}}_{{\rm{in}}-{\rm{c}}}={{\rm{F}}}_{{\rm{r}}}$$

where ${{\rm{\alpha }}}_{\mathrm{in}-{\rm{c}}}$ is the critical angle of inclination.

As shown in Fig. 1, the force equations of liquid droplets can be expressed by

7 $${{\rm{F}}}_{{\rm{Sx}}}+{\rm{mg}}\,{\sin {\rm{\alpha }}}_{{\rm{in}}}-{{\rm{F}}}_{{\rm{r}}}={{\rm{ma}}}_{{\rm{x}}}$$

8 $${{\rm{F}}}_{{\rm{Sz}}}-{\rm{mg}}\,{\cos {\rm{\alpha }}}_{{\rm{in}}}={{\rm{ma}}}_{{\rm{z}}}$$

From Equations 7 and 8 we know that the motion of droplet induced by acoustic waves on an inclined substrate could be affected by the acoustic streaming force, the gravity of droplet (droplet volume) and the inclination angle of the substrate. When increasing the acoustic streaming force, the speed of motion will increase. According to Equation 4 it implies that the speed of droplet motion is square of wave amplitude^14^. There is a linear relationship between the amplitude and the input power on the SPT^12^. Thereby, the speed of droplet motion is square of the input power on SPT. That is to say, the larger the input power on the SPT, the higher the speed of the droplet motion. In consequence the tip curvature will be larger, and the peals are easier to be emitted by the receding end.

Theory of Snoeijer *et al*.^20^ shows that the one-dimensional equation for curved contact line at the rear of the droplet can be described by the following equation:

9 $${\rm{h}}^{\prime\prime\prime} +\frac{1}{\tilde{{\rm{R}}}\tilde{{\rm{x}}}}(\frac{{\rm{h}}}{\tilde{{\rm{x}}}}-{\rm{h}}^{\prime} )=\frac{3\tilde{{\rm{Ca}}}}{{{\rm{h}}}^{2}},$$

where *h* represents the shape of the liquid-gas interface. $\tilde{{\rm{R}}}={\rm{R}}\,\tan \,{{\rm{\theta }}}_{{\rm{eq}}}$ and $\tilde{{\rm{Ca}}}={\mathrm{Ca}/\tan }^{3}\,{{\rm{\theta }}}_{{\rm{eq}}}$, here R is radius of curvature and ${{\rm{\theta }}}_{\mathrm{eq}}$ is the equilibrium contact angle. $\tilde{{\rm{x}}}={\rm{x}}\,\tan \,{{\rm{\theta }}}_{{\rm{eq}}}$ is the scaling the x direction with respect to the equilibrium contact angle.

Equation 9 predicts that with increasing the $\widetilde{\mathrm{Ca}}$, the macroscopic contact angle first decreases continuously without significant changes of the contact line curvature^20^. Approaching the critical speed of dewetting, when the contact angle further decreases, the contact line curvature will dramatically increase^20^. When the radius of curvature reaches a threshold value, the pearls will separated from the droplet. The experimental results in ref.^19^ demonstrates that the radius of curvature *R* does not extend below 80 μm, around which a cusp is formed at the rear of the droplet and pearls are emitted^19^.

## Electronic supplementary material

Supplementary Information
