# Supplementary material for: Pearls in running drops on an inclined glass substrate excited by Lamb waves
Source: Sci Rep. 2017 Oct 26;7:14164. doi: 10.1038/s41598-017-14662-9 (PMC5658407; doi:10.1038/s41598-017-14662-9)
Supplement: Supplementary file 1 — Supplementary Information [file 41598_2017_14662_MOESM1_ESM.pdf]

Supplementary Information

Pearls in running drops on an inclined glass substrate excited by Lamb waves

Wei Liang[1*], Sabrina Tietze[2]

[1]*Automotive Engineering College, Shanghai University of Engineering Science, Shanghai 201620, China*

[2]*Institute of Sensor and Actuator Technology, Coburg University of Applied Sciences and Arts, Coburg 96450, Germany*

**Correspondence to W.L.(wei.liang@live.de)*

I. Pearls generation

When the input power on the SPTs is immediately on, the velocities at liquid-solid interface are changing gradually except the positions around droplet's advancing and receding ends (Supplemental Figure S1).[12] Because of the inertia, pearls could be most probably left in the grooves (Supplemental Figure S2) around the positions of droplet's advancing and receding ends of initial state.

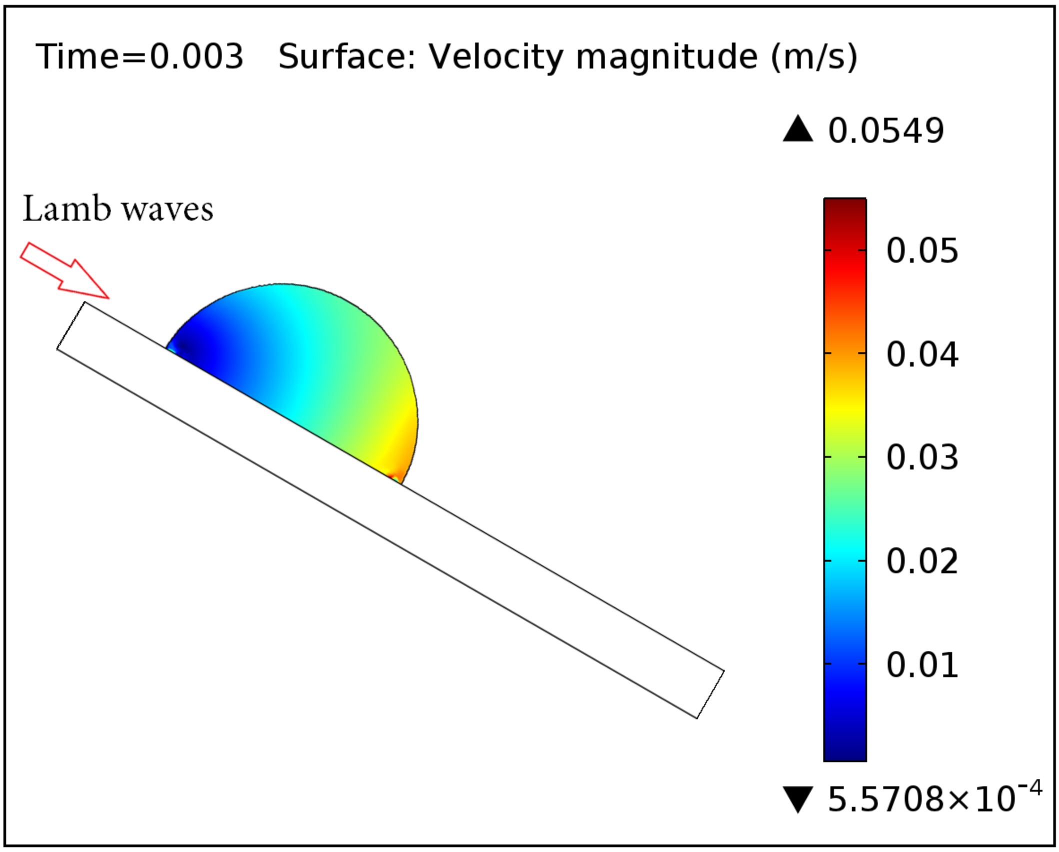

Supplemental Figure S1: Numerical results showing the (30 µl droplet at a Lamb wave amplitude of 9 nm). At first the velocities around the positions of the advancing and receding edges of droplet change suddenly.

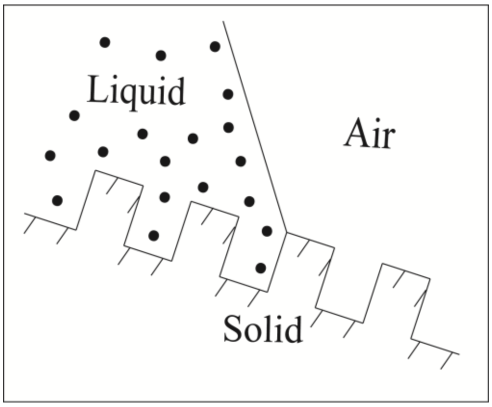

Supplemental Figure S2: Schematics of configuration of liquid droplet on the surface
